# COL12A1 Acts as a Novel Prognosis Biomarker and Activates Cancer-Associated Fibroblasts in Pancreatic Cancer through Bioinformatics and Experimental Validation

**DOI:** 10.3390/cancers15051480

**Published:** 2023-02-26

**Authors:** Yao Song, Lei Wang, Kaidong Wang, Yuhua Lu, Pengcheng Zhou

**Affiliations:** 1 Department of Radiotherapy and Oncology, Affiliated Hospital of Nantong University, Nantong 226001, China; 2 Department of Radiation Oncology, Tenth People’s Hospital Afflfliated to Tongji University, Shanghai 200072, China; 3 Department of General Surgery, Affiliated Hospital of Nantong University, Nantong 226001, China; 4 Department of Surgery, Medical School, Southeast University, Nanjing 210009, China

**Keywords:** pancreatic cancer, cancer-associated fibroblasts, COL12A1, TCGA, prognosis

## Abstract

**Simple Summary:**

Cancer-associated fibroblasts (CAFs) are key stromal cells in the tumor microenvironment (TME) that play a crucial role in tumor progression in pancreatic cancer. Thus, uncovering the key genes involved in CAF progression and determining their prognostic value is critically important. Analysis of The Cancer Genome Atlas (TCGA) dataset and investigation of our clinical tissue samples indicated that COL12A1 expression was aberrantly highly expressed in pancreatic cancer. COL12A1 was mainly expressed in CAFs but not in tumor cells. The knocking down of COL12A1 decreased the proliferation and migration of CAFs and down-regulated the expression of CAF activation markers. The cancer-promoting effect was reversed with COL12A1 knockdown. These findings indicate that COL12A1 acts as a novel prognosis biomarker and provides new opportunities for TME-targeted therapies in pancreatic cancer.

**Abstract:**

Pancreatic cancer remains one of the most challenging malignancies to date and is associated with poor survival. Cancer-associated fibroblasts (CAFs) are key stromal cells in the tumor microenvironment (TME) that play a crucial role in tumor progression in pancreatic cancer. Thus, uncovering the key genes involved in CAF progression and determining their prognostic value is critically important. Herein, we report our discoveries in this research area. Analysis of The Cancer Genome Atlas (TCGA) dataset and investigation of our clinical tissue samples indicated that COL12A1 expression was aberrantly highly expressed in pancreatic cancer. Survival and COX regression analyses revealed the significant clinical prognostic value of COL12A1 expression in pancreatic cancer. COL12A1 was mainly expressed in CAFs but not in tumor cells. This was verified with our PCR analysis in cancer cells and CAFs. The knocking down of COL12A1 decreased the proliferation and migration of CAFs and down-regulated the expression of CAF activation markers actin alpha 2 (ACTA2), fibroblast activation protein (FAP), and fibroblast-specific protein 1 (FSP1). Meanwhile, the interleukin 6 (IL6), CXC chemokine Ligand-5 (CXCL5), and CXC chemokine Ligand-10 (CXCL10) expressions were inhibited, and the cancer-promoting effect was reversed by COL12A1 knockdown. Therefore, we demonstrated the potential prognostic and target therapy value of COL12A1 expression in pancreatic cancer and elucidated the molecular mechanism underlying its role in CAFs. The findings of this study might provide new opportunities for TME-targeted therapies in pancreatic cancer.

## 1. Background

Pancreatic ductal adenocarcinoma (PDAC) is difficult to treat and has a 5-year survival rate of less than 5% [1]. As the early diagnosis of pancreatic cancer is difficult, 80% of patients are diagnosed in the advanced stage of the disease or have locally invasive tumors. Only 20% of patients have the opportunity for curative resection. These patients have an overall 5-year survival rate of 10–25% [2,3]. Moreover, local recurrence or distant metastasis is common, even in patients who have undergone surgery. In such cases, adjuvant therapy consisting of chemotherapy and/or radiation therapy is necessary. However, the outcomes of combined chemotherapy and radiotherapy remain poor [4]. Therefore, the identification of molecular prognostic biomarkers and targeted molecular therapies are essential to improve the outcomes of PDAC patients.

The extracellular matrix (ECM) performs a crucial role in tumor progression invasion and chemo-resistance in pancreatic cancer [5]. The most abundant protein component in the ECM is collagen, which can directly bind to the cancer cell receptors discoidin domain receptor 1 (DDR1) and discoidin domain receptor 2 (DDR2), regulate immune cell infiltration, and TGFβ expression indirectly associated with the cancer cell to induce tumor growth and metastasis [6,7]. Thirty-two types of collagens have been identified in ECM [8]. The different types of collagens expressed in PDAC have critical roles in cancer genesis and progression. Even some types of collagens have a prognosis value for PDAC patients as confirmed with serum, tissues, or bioinformatic analyses [9,10,11,12].

COL12A1, a member of the major collagen family of Fibril-Associated Collagens (FACIT) collagens, assumes a key role in tumor growth [6]. High COL12A1 expression has been correlated with poor survival and cancer metastasis in gastric cancer and colon cancer [13,14]. Bioinformatic analysis indicated COL12A1 has a prognosis effect in pancreatic cancer [15,16,17,18], but the mechanism underlying the effect of COL12A1 in PDAC progression is still unelucidated.

In this study, using the Gene Expression Omnibus (GEO) dataset, we found the pathway of differentially expressed genes (DEGs) that mainly focuses on the ECM receptor interaction and collagen catabolic process. The collagen family was focused on finding the key genes associated with pancreatic cancer progression. Analysis of the TCGA dataset implied that COL12A1 has an important role in pancreatic cancer prognosis. CAFs that expressed COL12A1 make a crucial contribution to PDAC genesis and progression. This was explored with bioinformatic analysis and validated using in vitro and in vivo experiments. The findings of this study might provide new opportunities for TME-targeted therapies in pancreatic cancer.

## 2. Methods

### 2.1. Data Collection

RNA sequencing and the related clinical data of TCGA and Genotype-Tissue Expression (GTEx) were downloaded from the UCSC XENA website (https://xenabrowser.net/datapages/, accessed on 5 December 2021). GSE16515, GSE15471, GSE60979, GSE62452, GSE71989, and GSE91035 gene expression profiles were retrieved from the GEO database (https://www.ncbi.nlm.nih.gov/geo/, accessed on 5 December 2021) in microarray platform (GLP570). These data were analyzed using the Affymetrix Human Genome U133 Plus 2.0 Array (transcript (gene) version; Santa Clara, CA, USA).

### 2.2. Identification of DEGs

After downloading the datasets for GSE16515, GSE15471, GSE60979, GSE62452, GSE71989, and GSE91035, the GEO2R online tool was used to identify DEGs. Tumor and normal tissues were selected to evaluate gene expression. The threshold value for the screening of DEGs was *p* < 0.05 and |log fold-change| > 1. The Online Venn diagram tool was used to visualize the DEGs in the six data sets.

### 2.3. GO Enrichment and KEGG Pathway Analysis of DEGs

The DAVID online tool (http://david.ncifcrf.gov/, version 6.8, accessed on 19 December 2019) [19] was used to check gene function (GO) and Kyoto Encyclopedia of Genes and Genomes (KEGG) pathways. GO describes genes in terms of their biological process (BP), molecular function (MF), and cellular component (CC) [20]. The KEGG pathway was used to check the indicated genes, including their reference pathways [21]. *p* < 0.05 was considered to indicate statistical significance.

### 2.4. Expression Analysis

GEPIA (http://gepia.cancer-pku.cn/detail.php, accessed on 19 December 2019) is an interactive web server for analyzing the RNA sequencing expression data of 9736 tumors and 8587 normal samples from the TCGA and GTEx projects. The GEPIA expression module was used to visualize mRNA expression in TCGA combined with GTEx. The Oncomine database, the largest oncogene chip database, was used to demonstrate the COL12A1 mRNA expression level difference between tumors and normal tissues. Additionally, we used the R package “ggplot2” to explore the relationship between COL12A1 mRNA expression and clinical parameters in TCGA. The Clinical Proteomic Tumor Analysis Consortium (CPTAC) dataset was used for COL12A1 protein expression analysis. The Human Protein Atlas (HPA) database (http://v13.proteinatlas.org/, accessed on 19 December 2019) is designed to map all the human proteins in cells, tissues, and cancers. It was used to demonstrate the differential expression of COL12A1 protein using immunohistochemistry staining in normal and cancer tissues. Tumor Immune Single-cell Hub (TISCH) is a scRNA-seq database (http://tisch.comp-genomics.org/home/, accessed on 21 May 2022) focusing on TME. In the “dataset” and “gene” modules, we visualized the expression levels of COL12A1 at the single-cell level in the pancreatic cancer RA001160 and GSE111672 datasets.

### 2.5. Survival, Prognosis, and Diagnostic Value Analysis

We evaluated the association of COL12A1 expression with overall survival (OS) using the GEPIA survival module. In addition, the R packages “survminer” and “survival” were used to visualize the COL12A1 expression, Disease Specific Survival (DSS), and Progress Free Interval (PFI). The log-rank test was used to compare differences in survival between the low and high levels of COL12A1 groups using the R package “ggrisk”. The R package “timeROC” was used to compare the predictive accuracy of COL12A1 mRNA. We established a nomogram combining COL12A1 expression and key clinical factors to predict the 1-, 3-, and 5-year survival of pancreatic cancer patients using the R packages “rms” and “survival”. Additionally, we conducted a calibration analysis to check the nomogram.

### 2.6. Gene Mutation and Methylation Analysis

Mutation analysis was performed on the cBioportal online web (http://www.cbioportal.org, accessed on 21 May 2022). Gene methylation analysis was performed on GSCA: Gene Set Cancer Analysis online web (http://bioinfo.life.hust.edu.cn/GSCA/, accessed on 21 May 2022) and UALCAN.

### 2.7. Functional Enrichment Analysis

To explore the abnormal changes in downstream pathways caused by the enhanced expression of COL12A1, we identified DEGs between pancreatic cancer samples with COL12A1 high and low mRNA expression based on the TCGA data using the R packages “DESeq2” and “ggplot2”. To further clarify the potential mechanisms of COL12A1 in pancreatic cancer progression, GO and KEGG enrichment was performed to predict the functions and pathways of the COL12A1-related DEGs using the R package “clusterProfiler”. In addition, we analyzed some important pathways involved in cancer using the R package “GSVA”, choosing parameter as method = ‘ssGSEA’. The correlation between genes and pathway scores was analyzed using Spearman correlation.

### 2.8. Cell Infiltration Analysis

Tumor Immune Estimation Resource (TIMER) is a comprehensive resource for the systematical analysis of immune infiltrates across diverse cancer types (http://timer.comp-genomics.org/, accessed on 1 April 2022). CAFs play a key role in the development and maintenance of the stromal cancer compartment, mediating an increase in the synthesis of the extracellular matrix [22]. To explore the correlation between COL12A1 expression and cancer-associated fibroblast (CAF) infiltrates, we applied the immune gene module and selected the CAFs for analysis. The correlation between COL12A1 expression and the cell markers of CAFs was also analyzed.

### 2.9. Cell Lines, Patients, and Specimens

PANC-1, BxPC-3, CFPAC-1, PATU-8988, ASPC-1, and MIA PaCa-2 were obtained from Procell. PANC-1, PATU-8988, and HPDE6-C7 were cultured with DMEM (Gibco) supplemented with 10% FBS (Gibco) and 1% penicillin-streptomycin (Gibco). ASPC-1 was cultured with RPMI-1640 (Gibco) supplemented with 10% FBS (Gibco) and 1% penicillin-streptomycin (Gibco). CFPAC-1 was cultured with IMDM (Gibco) supplemented with 10% FBS (Gibco) and 1% penicillin-streptomycin (Gibco). MIA PaCa-2 cells were cultured with DMEM (Gibco) supplemented with 10% FBS (Gibco), 5% HS (Gibco), and 1% penicillin-streptomycin (Gibco). To measure COL12A1 expression, 31 pairs of fresh pancreatic cancer tissues and matched para-cancer tissues were obtained between November 2019 and November 2021 at the Affiliated Hospital of Nantong University. The study protocol was approved by the Human Ethics Review Committees of Affiliated Hospital of Nantong University (approval no. 2019-L034).

### 2.10. Culture and Transfection of CAFs

CAFs were isolated from fresh pancreatic cancer tissues following the method described by Bachem et al. [17]. Pancreatic cancer tissues were obtained surgically and cut into small pieces and cultured in DMEM (Gibco) plus 10% FBS (Gibco) and 1% penicillin-streptomycin (Gibco) in T25 flasks. The medium was changed every three days. After 7–10 days, CAFs migrated out of the tissues. The cells were maintained in a humidified incubator at 37 °C in an atmosphere of 5% CO_2_. All resected tissues were postoperatively diagnosed with pancreatic cancer. All patients provided written informed consent, and the Ethics Committee of the Affiliated Hospital of Nantong University approved this study. CAFs were identified by the detection of the CAF-specific markers ACTA2, FAP, and FSP1 with immunofluorescence. CAFs at the logarithmic growth phase were digested and seeded into a 6-well plate. When cell confluence reached 60–70%, CAFs were transfected with siRNA NC and siRNA COL12A1 (GCAAUAAACACCUUCCCUUTT) using Lipofectamine 2000 reagents.

### 2.11. RNA Extraction and Quantitative Real-Time PCR

Total RNA was extracted from cells or tissues using Trizol reagent (Invitrogen, Carlsbad, CA, USA) following the manufacturer’s protocol. The extracted RNA was reverse transcribed into complementary DNA (cDNA) following the instructions of the Reverse Transcription Kit (Takara, Kusatsu, Japan). Thereafter, the cDNA was subjected to real-time PCR using the SYBR Green PCR kit (Takara, Kusatsu, Japan) and the Step One instrument (Applied Biosystems, Carlsbad, CA, USA). The primers used in the study were provided in Table 1.

### 2.12. EdU, Wound Healing, Transwell, and Clone Formation Assay

For the EdU assay, CAFs were seeded into a 24-well plate. Each well was incubated with EdU medium for 3 h and then fixed with 4% paraformaldehyde. The cells were further incubated with EdU regent in the dark for 30 min. Finally, cells were incubated with Hoechst 3334 for 10 min. Cells stained with EdU and with Hoechst 33342 were counted. EdU positive rate (%) = the number of EdU cells/the number of total cells × 100%. For the wound healing assay, CAFs were seeded in 12-well plates. When the confluent monolayer was formed, the cells were starched with a sterile 200 µLpipette tip to create a wound gap. The medium was replaced with an FBS-free medium and cultured for another 24 h. For the transwell assay, an 8 μm transwell chamber (Corning, kennebunk, ME, USA) was used. A CAF suspension (100 µL, 4 × 10^4^ cells) was added to the upper chamber and a medium containing 10% FBS was added to the lower chamber. After incubation at 37 °C for 18 h, the cells were fixed with 4% paraformaldehyde and stained with crystal violet. Eight fields of view were randomly selected, and the cell number was counted under the microscope. For the clone formation assay, the conditional medium of CAFs transfected with siNC and siCOL12A1 was collected, respectively. Pancreatic cancer cells PANC-1 were seeded into 12-well plates at a density of 500 cells/well with different conditional medium. Finally, the cells were fixed with 4% paraformaldehyde and stained with crystal violet. The number of colonies in each well was counted.

### 2.13. Western Blotting and Immunofluorescence

For western blotting, total proteins were extracted from cells using radioimmunoprecipitation assay lysis buffer (SolarBio Science & Technology Co., Ltd., Beijing, China). Protein concentration was quantified using a bicinchoninic acid (BCA) kit (Beyotime Biotechnology, Nantong, China). The protein was separated using polyacrylamide gel electrophoresis and electrotransferred onto polyvinylidene fluoride (PVDF) membranes using the wet transfer method. The membrane was blocked with NcmBlot blocking buffer (New cell & Molecular Biotech Co., Ltd., Suzhou, China) for 10 min and incubated with primary antibodies against ACTA2 (1:1000, Servicebio, Wuhan, China), FAP (1:500, Beyotime Biotechnology, Nantong, China), and FSP (1:1000, Peoteintech, Wuhan, China) at 4 °C overnight. Next, the membrane was incubated with horseradish peroxidase (HRP)-labeled goat anti-rabbit IgG (1:10,000, Biosharp, Hefei, China) at room temperature for 1 h and then developed. For immunofluorescence, cells were seeded onto slides and fixed with 4% paraformaldehyde. Then, they were incubated with primary antibody against ACTA2 at 4 °C overnight. Next, cells were incubated with a secondary antibody goat anti-rabbit Alexa Fluor 488 IgG (1:200, Servicebio, Wuhan, China) at room temperature for 1 h in the dark. Finally, the cells were stained with Hoechst 33342. The slides were visualized under a confocal microscope.

### 2.14. Statistical Analysis

We performed univariate Cox proportional hazard analysis to identify hub genes significantly related to patient survival (*p* < 0.05). Genes that significantly correlated with patient survival in univariate analysis were included in multivariate Cox regression analysis. All data were statistically analyzed using GraphPad Prism 8.0 (GraphPad Software, La Jolla, CA, USA), and all experiments were independently repeated at least thrice. Measurement data were expressed as mean ± standard deviation (SD). Two groups of data were compared using an independent sample test-test. A value of *p* < 0.05 was regarded as statistically significant.

## 3. Results

### 3.1. DEGs Were Identified in Pancreatic Cancer

**Figure 1:**
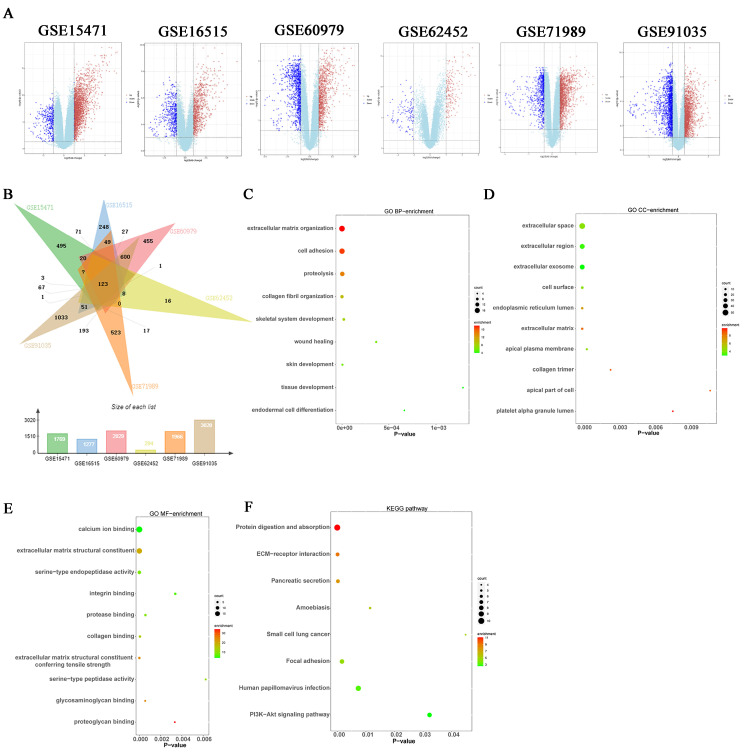
DEGs and their functions in GSE15471, GSE16515, GSE60979, GSE62452, GSE71989, and GSE91035. (**A**) Volcano plot of DEGs between pancreatic cancer tissues and normal pancreas tissues in the GSE15471, GSE16515, GSE60979, GSE62452, GSE71989, and GSE91035 datasets. (**B**) Venn diagram of overlapping 123 DEGs from the GSE15471, GSE16515, GSE60979, GSE62452, GSE71989, and GSE91035 datasets; (**C**) GO (Biological process) analysis of the overlapping DEGs in pancreatic cancer. (**D**) GO (Cellular component) analysis of the overlapping DEGs in pancreatic cancer. (**E**) GO (Molecular function) analysis of the overlapping DEGs in pancreatic cancer. (**F**) KEGG pathway analysis of the overlapping DEGs in pancreatic cancer.

Six datasets, namely GSE16515, GSE15471, GSE60979, GSE62452, GSE71989, and GSE91035, were obtained from the National Center for Biotechnology Information GEO database, which contains data for pancreatic cancer and normal tissue samples. A total of 1769 DEGs in GSE15471, 1277 DEGs in GSE16515, 2029 DEGs in GSE60979, 294 DEGs in GSE62452, 1966 DEGs in GSE71989, and 3020 DEGs in GSE91035 were identified using the criteria *p* < 0.05 and |log fold-change| > 1 (Figure 1A,B). The Venn diagram shows that 123 genes were regulated (Figure 1B).

### 3.2. Enrichment Analysis of DEGs

The DAVID online tool was used to analyze the biological function of overlapping DEGs. GO in terms of BP, CC, and MF of overlapping DEGs among the regulated genes were analyzed. The most extensive BP enrichment was observed during extracellular matrix organization and collagen fibril organization; CC enrichment was the highest in the extracellular space and extracellular region; MF enrichment was the highest in extracellular matrix structural constituents and calcium ion binding. The investigation of the signaling pathway of the overlapping DEGs revealed that the protein digestion, absorption, and ECM-receptor interaction were the most important KEGG pathways (Figure 1C–F).

### 3.3. Identification of the Key Gene, COL12A1

**Figure 2:**
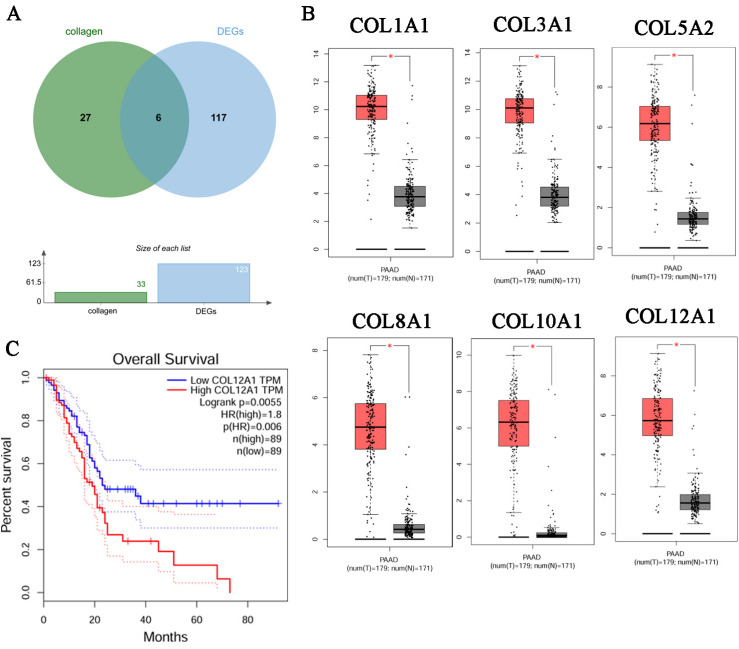
Identification of the key gene, COL12A1. (**A**) Venn diagram of overlapping 6 DEGs from 123 DEGs in the GSE15471, GSE16515, GSE60979, GSE62452, GSE71989, and GSE91035 datasets and 32 collagens. (**B**) Gene Expression Profiling Interactive Analysis (GEPIA) was performed to validate the expression of six hub genes in pancreatic cancer samples compared with normal samples. Red box was the cancer tissue group, gray was the normal tissue group, and asterisk represented *p* < 0.01. The dots represented expression in each sample. (**C**) Check the Over survival (OS) curves of COL12A1 in pancreatic cancer tissues in TCGA dataset. (*p* < 0.05).

We collected the genes common for DEGs and collagen family genes. The Venn diagram shows that six collagen genes, COL1A1, COL3A1, COL5A2, COL8A1, COL10A1, and COL12A1, were identified between 123 DEGs and 32 collagen genes (Figure 2A). The TCGA and GETx datasets indicated that COL1A1, COL3A1, COL5A2, COL8A1, COL10A1, and COL12A1 genes were expressed much higher in tumor tissues than in the normal ones (Figure 2B). The survival rate was much worse for patients with high COL12A1 expression than that in patients with low expression. However, this is not the case for COL1A1, COL3A1, COL5A2, COL8A1, and COL10A1 (Figure 2C and Figure S1).

### 3.4. Verification That COL12A1 Expression Was Much Higher in Tumor Tissues Than in Para-Cancer Tissues

**Figure 3:**
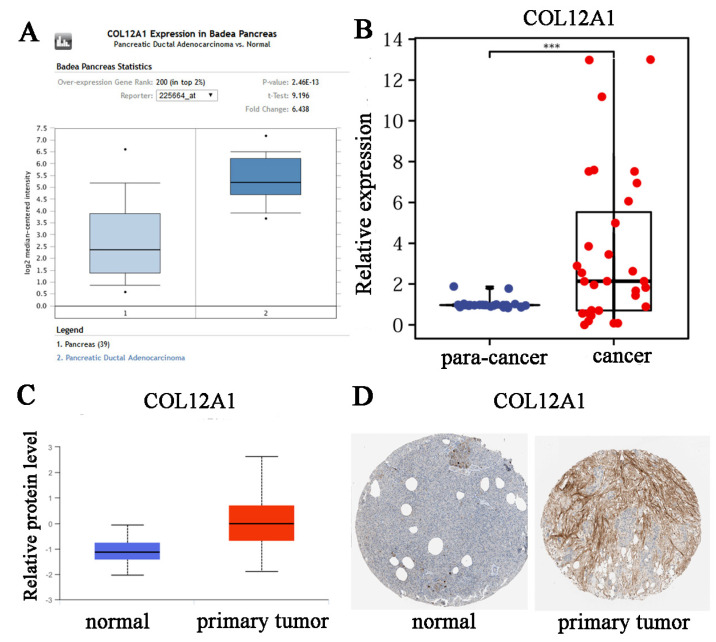
Verification that COL12A1 expression was much higher in tumor tissues than in para-cancer tissues. (**A**) Oncomine was performed to validate the expression of COL12A1 in pancreatic cancer samples compared with normal samples. (**B**) COL12A1 expression levels in 31 pairs of fresh pancreatic cancer tissues and their para-cancer tissues. (COL12A1/GAPDH). (**C**) Verified COL12A1 expression was much higher in tumor tissues than in normal tissues in the CPTAC datasets. (**D**) Immunohistochemistry of COL12A1 based on the Human Protein Atlas. ***, *p* < 0.001.

The Oncomine online tool indicated a higher COL12A1 expression in tumor tissues than that in normal tissues (Figure 3A). COL12A1 expression was much higher in cancer tissue than in para-cancer tissue as indicated by the qPCR results (Figure 3B). CPTAC and HPA indicated that the protein level of COL12A1 was significantly higher in tumor tissues than in normal tissues (Figure 3C,D).

### 3.5. Clinical Characteristics of COL12A1

**Figure 4:**
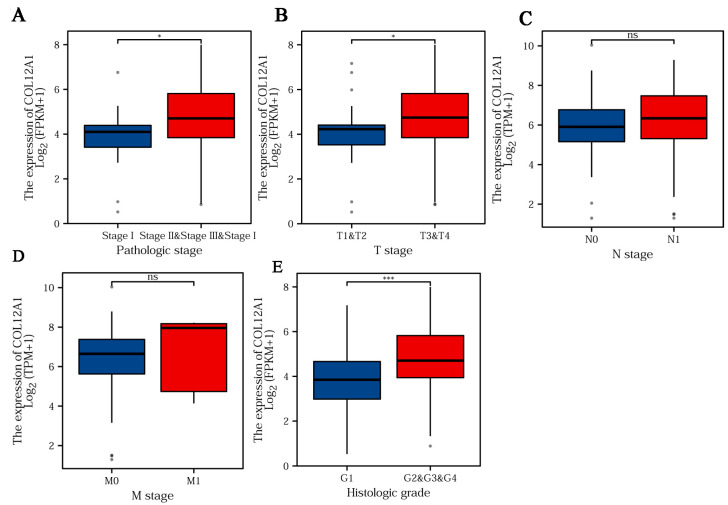
COL12A1 expression separated by different characters. (**A**) Pathological stages; (**B**) T stage; (**C**) N stage; (**D**) M stage; and (**E**) Histologic grade. *, *p* < 0.05, ***, *p* < 0.001, ns, non-significant.

**Figure 5:**
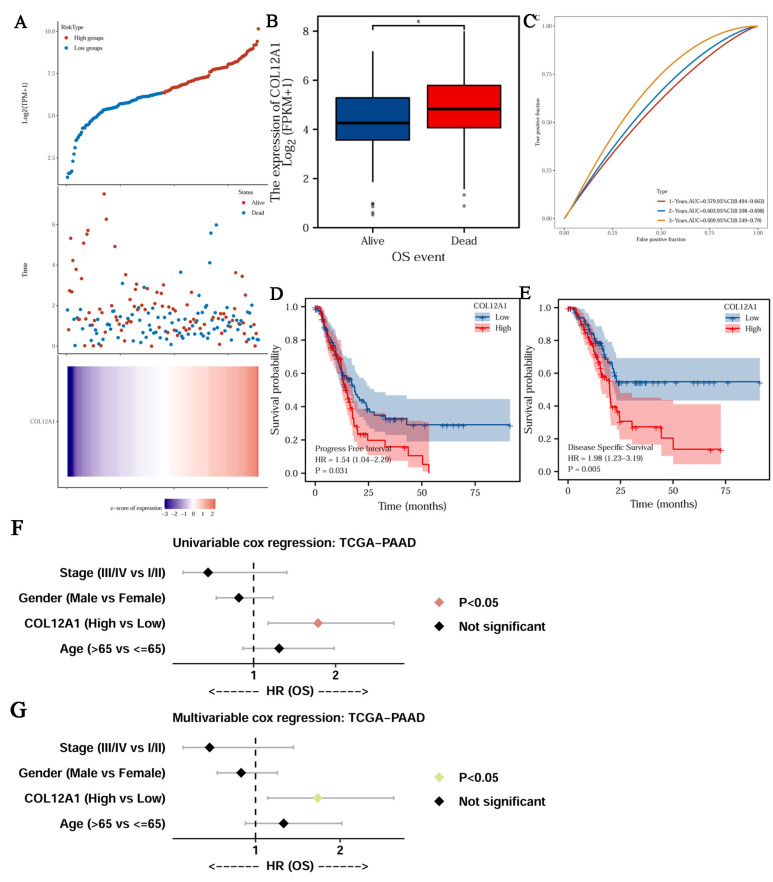
The clinical prognosis of COL12A1 in pancreatic cancer. (**A**) The dead and alive patients in low-COL12A1 expression people and high-COL12A1 expression people. (**B**) OS event in low-COL12A1 expression people and high-COL12A1 expression people. (**C**) Receiver operating characteristic (ROC) curve analysis and area under the curve (AUC) statistics were implemented to evaluate the capacity of COL12A1 to identify the prognosis in pancreatic cancer. (**D**) The progression-free interval (PFI) curve of COL12A1 in pancreatic cancer (*p* < 0.05). (**E**) Disease-specific survival (DSS) curve of COL12A1 in pancreatic cancer (*p* < 0.05). (**F**) Univariable analysis for the OS analysis in pancreatic cancer in the TCGA dataset. (**G**) Multivariate analysis for the OS analysis in pancreatic cancer analysis in the TCGA dataset. *, *p* < 0.05.

**Figure 6:**
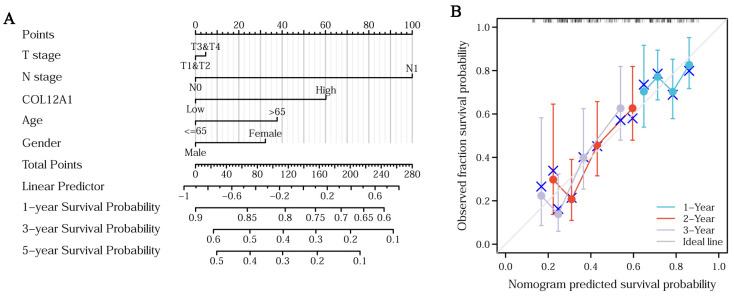
Nomogram analysis for pancreatic cancer over survival. (**A**) The nomogram analysis involving COL12A1 expression, age, gender, and the T and N stages to predict the prognosis in pancreatic cancer patients. (**B**) Predicted survival ability of this nomogram for 1, 2, and 3 years.

Much higher COL12A1 expression was observed in stages III/IV than in stages I/II (Figure 4A). The expression of COL12A1 was significantly associated with the T stage, higher in T3/4 than that in T1/2 (Figure 4B), but not in the N and M stages (Figure 4C,D). In histologic grades, II/III/IV, much higher COL12A1 expression was observed than that in grade I (Figure 4E) (histologic grade in TCGA means the numeric value to express the degree of abnormality of cancer cells; it is a measure of differentiation and aggressiveness). High COL12A1 expression was positively correlated with a worse prognosis (Figure 5A). The COL12A1 expression was much higher in dead patients than in alive people (Figure 5B). The ROC curve indicated that COL12A1 might be a predictor for the survival rate of pancreatic cancer patients. The area under the curve was 0.579 for 1-year survival, 0.603 for 2-year survival, and 0.669 for 3-year survival (Figure 5C). Moreover, based on the TCGA dataset, the progression-free interval (PFS) was much worse for patients with high COL12A1 expression than that for those with low expression (HR = 1.54, 1.04–2.29, *p* = 0.031) (Figure 5D). The disease-specific survival (DSS) was much longer in low-COL12A1 expression patients than that in high-expression ones (HR = 1.98, 1.23–3.19, *p* = 0.005) (Figure 5E). Univariable and multivariable cox regression analyses showed that the COL12A1 expression level was an independent determinant to predict the outcome of pancreatic cancer patients. (Figure 5F,G). COL12A1 expression was combined with age, gender, and the T and N stages to build a nomogram for OS prediction (Figure 6A). The nomogram is predictive of the OS for pancreatic cancer patients and demonstrates comparatively high accuracy, as shown by the calibration curves (Figure 6B).

### 3.6. Genetic Alterations and Mechanism of Hub Gene Regulation

We investigated the complex molecular properties of COL12A1 in PDAC tissues. The TCGA dataset was used to analyze genetic alterations, which were found to be 1.6% for COL12A1 in PDAC tissues. The COL12A1 methylation was negatively correlated to the COL12A1 mRNA expression in PDAC patients. However, the expression of methylated COL12A1 expression was much higher in tumor tissue than in normal tissue in the pancreas. In the pancreatic cancer tissues with P53 and KRAS mutation, COL12A1 expression is much higher than that in the wild-type pancreatic cancer tissues, as shown in Figure S2A–F.

### 3.7. COL12A1 Is Mainly Expressed in CAF and Correlated with Fibroblast Activation Protein Expression

**Figure 7:**
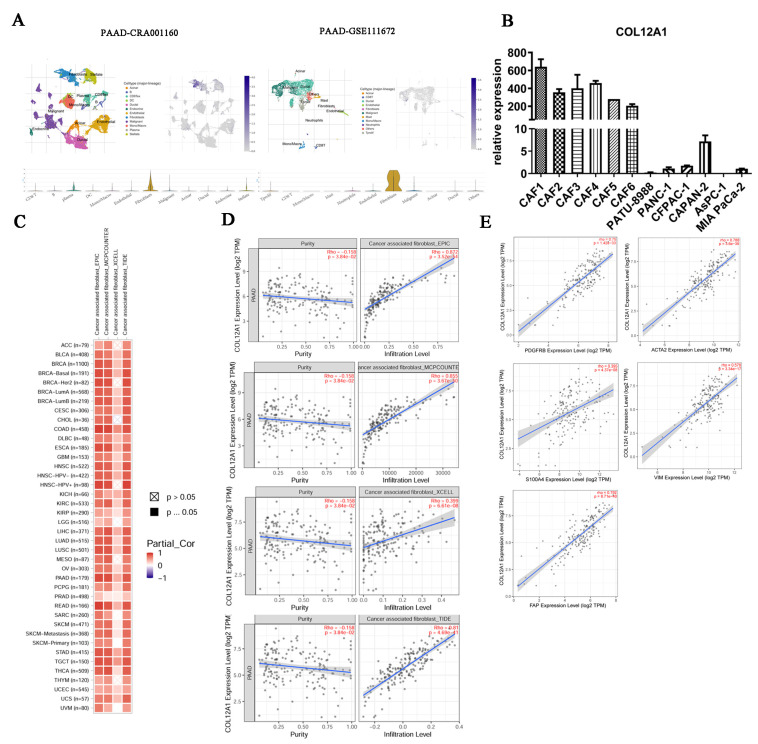
COL12A1 is mainly expressed in CAF and correlated with fibroblast activation protein expression. (**A**) TISCH checking indicated the major COL12A1-expressing cells in pancreatic cancer. (**B**) qPCR verified the COL12A1 expression in pancreatic cancer cell lines and cancer-associated fibroblast cells. (**C**) Relationship between COL12A1 and cancer-associated fibroblast infiltration in pan-cancer. (**D**) COL12A1 and cancer-associated fibroblast in pancreatic cancer infiltration were checked using the EPIC, MCPCOUNTE, XCELL, and TIDE methods. (**E**) Correlations between COL12A1 and fibroblast activation protein expression.

TISCH checking indicated that COL12A1 is primarily expressed in cancer-associated fibroblasts but not in tumor cancer cells or other immune cells in TME for pancreatic cancer (Figure 7A). The expression of COL12A1 was significantly higher in CAF than in tumor cells as evidenced using qPCR (Figure 7B). The different methods indicated COL12A1 was crucially related to cancer-associated fibroblast infiltration. Furthermore, COL12A1 expression correlated with the expression of the genes associated with fibroblast activation (Figure 7C–E).

### 3.8. The Enrichment Function for COL12A1

**Figure 8:**
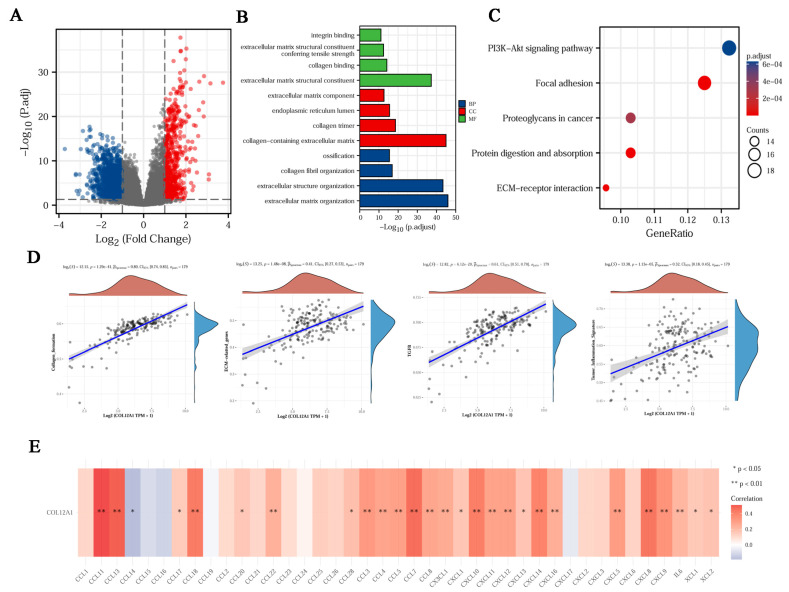
Enrichment function for COL12A1. (**A**) Volcano plot of DEGs between high and low COL12A1 expressions. (**B**) GO analysis of the DEGs based on the COL12A1 expression in pancreatic cancer. (**C**) KEGG analysis of the DEGs based on the COL12A1 expression in pancreatic cancer. (**D**) COL12A1 and associated pathway in high and low COL12A1 expressions. (**E**) Correlation of COL12A1 expression and chemokines in PDAC.

COL12A1 co-expression networks were studied using the TCGA database to verify the potential function of COL12A1 in tumor tissues. A total of 616 genes were significantly upregulated to COL12A1, and 1586 genes were significantly downregulated to COL12A1 (Figure 8A). LRRC53, TCP11X2, FGL1, C6orf58, and CALY represented the genes downregulated with COL12A1 expression. Conversely, EPYC, MAB21L2, CASP14, COL11A1, and APELA represented the genes upregulated with COL12A1 expression (Figure S3A,B). The most enriched BP, CC, and MF were the extracellular matrix structural constituents, collagen-containing extracellular matrix, and extracellular matrix organization. The investigation of the signaling pathway of the overlapping DEGs revealed that the PI3K/AKT pathway was the most important KEGG pathway (Figure 8B,C). Further, the pathway correlation results indicated that COL12A1 expression correlated with collagen formation, ECM-related genes, the TGF-β pathway, and the inflammation signature (Figure 8D). Finally, CO112A1 expression was positively correlated with 27 out of 41 chemokines (Figure 8E).

### 3.9. Knockdown of COL12A1 Reversed the Phenotype of CAFs, Inhibited Chemokines Expression, and Reversed the Promoting Effect on Pancreatic Cancer Cells

**Figure 9:**
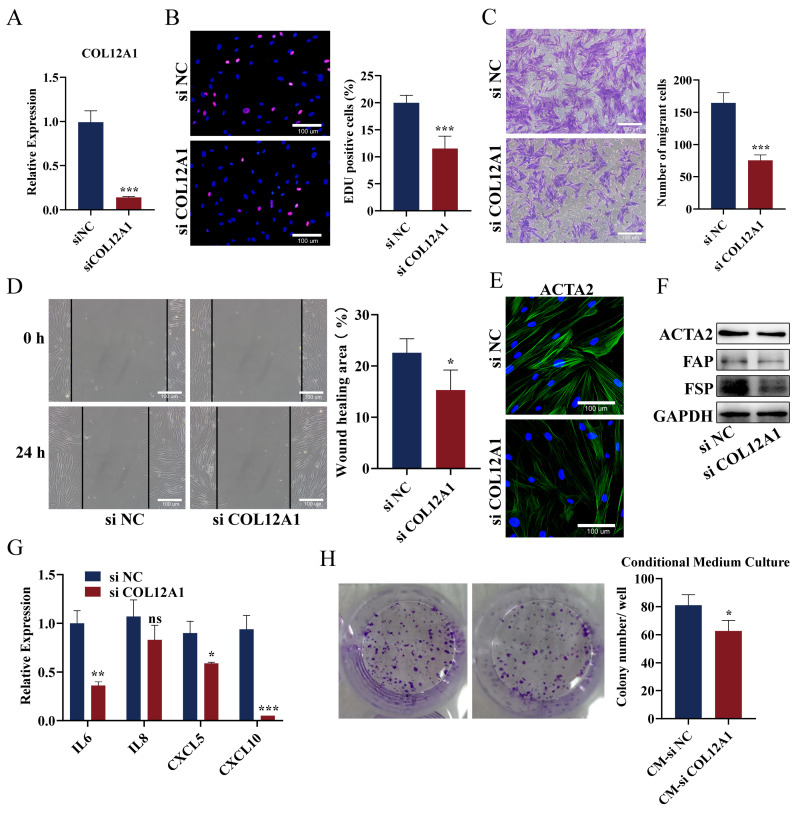
Verification of the function of COL12A1 in CAF and tumor cells. (**A**) COL12A1 mRNA expression in siCOL12A1- and siNC-transfected CAFs. (**B**) EDU assay indicated the proliferation ability of CAFs in the siCOL12A1 and siNC groups. (**C**) Transwell assay analyzed the CAFs migration after COL12A1 knockdown. (**D**) Wound healing checked the CAFs migration in siCOL12A1- and siNC-transfected CAFs. (**E**) Immunofluorescence intensity of ACTA2 and the number of ACTA2 fibers were checked after COL12A1 knockdown in CAFs. (**F**) Western blot demonstrated the protein level of ACTA2, FAP, and FSP expressions after COL12A1 knockdown in CAFs. (**G**) qPCR checked IL6, IL8, CXCL5, and CXCL10 expressions in siCOL12A1- and siNC-group CAFs. (**H**) Collected the supernatant from siCOL12A1- and siNC-group CAFs and then added it to incubate Panc-1 cells. Detected the clone formation of Panc-1 cells with conditional medium (*, *p* < 0.05, **, *p* < 0.01, ***, *p* < 0.001, ns, non-significant).

We used a small interfering RNA (siRNA) to knock down COL12A1 in CAFs and explored the potential bio-function. COL12A1 mRNA expression was significantly downregulated in CAFs transfected with siCOL12A1 compared with siNC (Figure 9A). The findings from the EDU assay indicated that COL12A1 knockdown inhibited the proliferation ability of CAFs (Figure 9B). Transwell migration and wound healing activities were compromised after COL12A1 knockdown (Figure 9C,D). In addition, the immunofluorescence intensity of ACTA2 and the number of ACTA2 fibers were reduced after COL12A1 knockdown in CAFs (Figure 9E). The western blot demonstrated that COL12A1 knockdown can decrease the protein level of ACTA2, FAP, and FSP (Figure 9F). Moreover, we observed a decrease in IL6, CXCL5, and CXCL10 expression in siCOL12A1 CAFs compared to that that in the siNC groups (Figure 9G). Importantly, the culture medium of CAFs treated with siNC or siCOL12A1 was collected. The two kinds of conditional medium were added into pancreatic cancer cell PANC-1. The colony formation assay results implied that COL12A1 knockdown reversed the promoting effect of CAF on PANC-1 cells (Figure 9H).

## 4. Discussion

The occurrence and development of the solid tumor are accompanied by the connective tissue hyperplasia reaction and the deposition and remodeling of the tumor matrix, which can lead to significant changes in the tumor microenvironment [23,24]. The change in cell polarity and loosening of the adhesion between cells in the tumor environment occurred through alterations in the composition and accumulation mode of the extracellular matrix, resulting in the promotion of tumor growth, invasion, and metastasis [25]. Abnormal extracellular matrix (ECM), as a major component of PDAC stroma, regulated malignant cell behavior, and induced tumor formation and progression [5].

In this study, 123 DEGs were selected in pancreatic cancer from 6 GEO datasets. For enrichment analysis of the overlapping 123 DEGs, the extracellular matrix structural constituent exhibited the most enrichment for molecular function, the extracellular matrix organization was one of the most enriched of the biological processes, and the extracellular space and the extracellular region experienced the highest enrichment for the cellular component. ECM-receptor interaction was one of the most important KEGG pathways for pathway signaling. These results indicated that the change in the ECM played a crucial role in pancreatic cancer genesis and development. Collagen, as the main component of the extracellular matrix, performs some key functions in the development of tumors [26]. Different collagen proteins played different roles in the occurrence and development of pancreatic cancer. Some can promote tumor metastasis, while others can inhibit tumor growth [9,12,27,28,29]. Hence, we hypothesized that certain collagens perform important roles in the occurrence and development of pancreatic cancer.

We crossed the DEGs with 32 genes in the collagen family. Six collagen family genes were used for prognostic analysis in pancreatic cancer tissue. Only the COL12A1 gene was found to be notably related to the survival and prognosis of pancreatic cancer, but the others had no significant association, as shown in Figure 1. Therefore, we focused on COL12A1 to explore its mechanism in the occurrence and development of pancreatic cancer. Thirty-one pairs of clinical specimens were used to verify that COL12A1 expression in pancreatic cancer tissue was significantly higher than that in para-cancerous tissue. The result indicated COL12A1 might be a diagnosis and prognosis biomarker in pancreatic cancer. Several published papers have already indicated that COL12A1 might be the key prognosis biomarker for pancreatic cancer. Ding and Chen et al. indicated that MMP14 and COL12A1 constituted the potential combination of prognostic biomarkers in pancreatic cancer based on bioinformatics analysis. Performing bioinformatic analysis, Jing and Chen indicated that COL12A1 was the potential prognosis biomarker in pancreatic cancer [14,15,16,17]. However, the aforementioned results are primarily based on large databases and are thus lacking in vitro and in vivo findings or real-world data. Simultaneously, the source and mechanism of COL12A1 secretion are not evident. Therefore, using bioinformatic analysis and experimental verification, we further explored the source of COL12A1 and investigated the mechanism of its involvement in tumor genesis and development.

CAFs are the main contributor to tumor fibrosis, through increasing the synthesis of ECM proteins such as collagens and cross-linking enzymes, which could create a tumorigenic fibrotic environment around the PDAC tumor. Tumor fibrosis was closely associated with pancreatic cancer progression and drug resistance [30,31,32]. In the current study, the single-cell analysis revealed that COL12A1 was mainly distributed in CAF cells but not in tumor cells and other adjacent cells. Furthermore, the results from our cell line analysis indicated that the COL12A1 expression in the extracted para-cancerous fibroblasts was significantly higher than that in pancreatic cancer cells. CIBERSORT, xCELL, TIDE, and EPIC analysis for the dataset indicated that COL12A1 was correlated with CAF infiltration. These results revealed that COL12A1 was mainly derived from CAFs in pancreatic cancer. This finding is consistent with the previously published results related to breast and colon cancers [33,34], which indicated that COL12A1 is mainly expressed in CAF cells. COL12A1 secreted by CAF can change type I collagen tissue, support the pre-invasion microenvironment of metastatic transmission, and promote organ regeneration. Furthermore, we verified that COL12A1 expression could induce CAF cells to express the fibro-activated proteins ACTA2, FAP, and FSP, enhancing the cell invasion and release of inflammatory factors. Simultaneously, COL12A1 could promote tumor cell growth in a paracrine manner.

A substantial amount of data has indicated that CAFs act not only as bystanders but are actively involved in the process of cancer initiation, progression, and metastasis. Hence, the CAFs can be considered as the tumor promoter in pancreatic cancer [35,36]. The most commonly exploited CAF biomarkers in PDAC are ACTA2, FAP, vimentin, FSP1, podoplanin (PDPN/gp38), and platelet-derived growth factor receptor alpha and/or beta (PDGFRa/b) [37]. Our results also indicated that the expression of COL12A1 positively correlated with PDGFRB, ACTA2, S100A4, VIM, and FAP expressions at the mRNA level in the TCGA dataset. The CAFs in pancreatic cancer demonstrated heterogeneity for three types: myofibroblastic CAFs (myCAFs), inflammatory CAFs (iCAFs), and antigen-presenting CAFs (apCAFs). These three types can be separated by the biomarkers of myCAF, which exhibits the high expression of ACTA2, FAP, and TGF-β, and low expression of IL6, which requires direct interaction with cancer cells in PDAC and might be associated with tumor progression. ICAF, with low ACTA2 expression and high IL6 secretion, can be activated by paracrine factors secreted from tumor cells. However, the location of ICAF was found to be far from the tumor cells and myCAF. Moreover, apCAFs, expressed by MHC class II family genes, exhibit an antioxidant response. The activation and location of the apCAFs need further exploration to be fully understood [35,38,39,40]. Several studies have indicated that the TGF-β pathway can induce fibroblast transformation into myCAF and inhibit iCAF transformation. The findings of our study involving bioinformatic analysis indicated that COL12A1 expression was positively associated with the TGF-B pathway. The knockdown of COL12A1 inhibited ACTA2, FAP, and FSP1 expressions, which might indicate that COL12A1 expression is associated with myCAF transformation and activation.

In the CAF subtype analysis, FAP + CAF could induce PDAC progression and indicate worse survival. However, the function of aSMA + CAF was controversial. Athleen. et al. used single-cell RNA sequencing and several genetic mouse models to show that the depletion of FAP + CAF leads to increased survival, but depleting αSMA + CAFs resulted in decreased survival. However, depleting IL6 in a-SMA + CAF could increase gemcitabine sensitivity for the PDAC mice through T cell regulation [41]. Furthermore, Yurina et al., using 215 under-treatment PDAC patient samples, reported that aSMA-dominant and FAP-dominant fibroblast-rich stroma indicated poor prognosis [42]. Sun et al. used in vitro and in vivo experimental results to indicate that CXCR2/CXCL3 enhanced PDAC metastasis by inducing CAF toward myoCAF transformation and upregulated α-SMA expression [43]. Herein, we also found that COL12A1 knockdown can suppress CAF invasion and inhibit CAF biomarker aSMA and FAP expressions and cytokine IL6, chemokine CXCL5, and CXCL10 expression. The bioinformatics analysis also indicated that COL12A1 expression was highly associated with cytokine and chemokine expressions, such as IL6, IL8, CXCL5, and CXCL10 expressions. Furthermore, the colony formation of tumor cells can be suppressed when cultured in a conditional medium, which was collected from siCOL12A1 CAFs but not in the one collected from the group with siNC CAFs. The aforementioned result indicated that CAF expresses COL12A1 and could enhance tumor growth in a paracrine manner through increasing cytokine or chemokine secretion. The above results indicated that COL12A1 might provide new opportunities for TME-targeted therapies in pancreatic cancer. However, the exact mechanism of how COL12A1 effect CAF transformation as well as which subtype CAF was changed and induced tumor growth still not fully elucidated. The key factor that caused the COL12A1-induced tumor growth needs further exploration.

Despite many useful inputs, there are several limitations in our research. First, the CAF collected herein were combined with myCAF, iCAF, and apCAF, which did not separate. In further studies, we need to investigate which part of CAFs expressed COL12A1, and thus plays an important role in tumor growth. Second, the prognosis value and pathways were primarily considered by the dataset but not our own clinical samples. Hence, it is possible that in a further study, we need to check OS for our clinical sample for further validation of the results.

In this research, we systematically analyzed the mechanism involved in using COL12A1 as a therapy target and prognosis biomarker for pancreatic cancer. Our study demonstrated the potential diagnostic and prognostic value of COL12A1 expression in pancreatic cancer and elucidated the possible molecular mechanism underlying its role in promoting the development of pancreatic cancer in CAFs. These findings indicate that COL12A1 acts as a novel prognosis biomarker and provides new opportunities for TME-targeted therapies in pancreatic cancer.

## 5. Conclusions

Our study demonstrated the potential diagnostic and prognostic value of COL12A1 expression in pancreatic cancer and elucidated the molecular mechanism underlying its role in CAFs and promoting the development of pancreatic cancer. These findings might provide new opportunities for TME-targeted therapies in pancreatic cancer.

## Figures and Tables

**Table 1:** The primer sequence.

Primer	Sequence
CXCL8-F	TGGCAGCCTTCCTGATTTCT
CXCL8-R	TTTCTGTGTTGGCGCAGTGT
IL6-F	AGTGGCTGCAGGACATGACAA
IL6-R	CAATCTGAGGTGCCCATGCTA
CXCL5-F	GAGAGAGCTGCGTTGCGTTT
CXCL5-R	TTCAGGGAGGCTACCACTTC
CXCL10-F	CCTCTCTCTAGAACTGTACGCT
CXCL10-R	TCAGACATCTCTTCTCACCCT
COL12A1-F	TATTGTGTTCTTGACTGATGCCTCCTG
COL12A1-R	AGACTTGACCTCATCGCTGTATTGC
GAPDH-F	GCCAAAAGGGTCATCATCTC
GAPDH-R	TGAGTCCTTCCACGATACCA

## Data Availability

All data generated or analyzed during this study are included in this published article.

## Supplementary Materials

The following supporting information can be downloaded at: https://www.mdpi.com/article/10.3390/cancers15051480/s1. Figure S1: Survival rates for COL1A1, COL3A1, COL5A2, COL8A1, and COL10A1 high and low expressions in pancreatic cancer; Figure S2: Genetic Alterations and Mechanism of COL12A1 Regulation; Figure S3: Represented genes correlated with COL12A1 expression.
