# Supplementary material for: COL12A1 Acts as a Novel Prognosis Biomarker and Activates Cancer-Associated Fibroblasts in Pancreatic Cancer through Bioinformatics and Experimental Validation
Source: Cancers (Basel). 2023 Feb 26;15(5):1480. doi: 10.3390/cancers15051480 (PMC10000532; doi:10.3390/cancers15051480)
Supplement: Supplementary file 1 [file cancers-15-01480-s001.zip › cancers-2240265-supplementary.pdf]

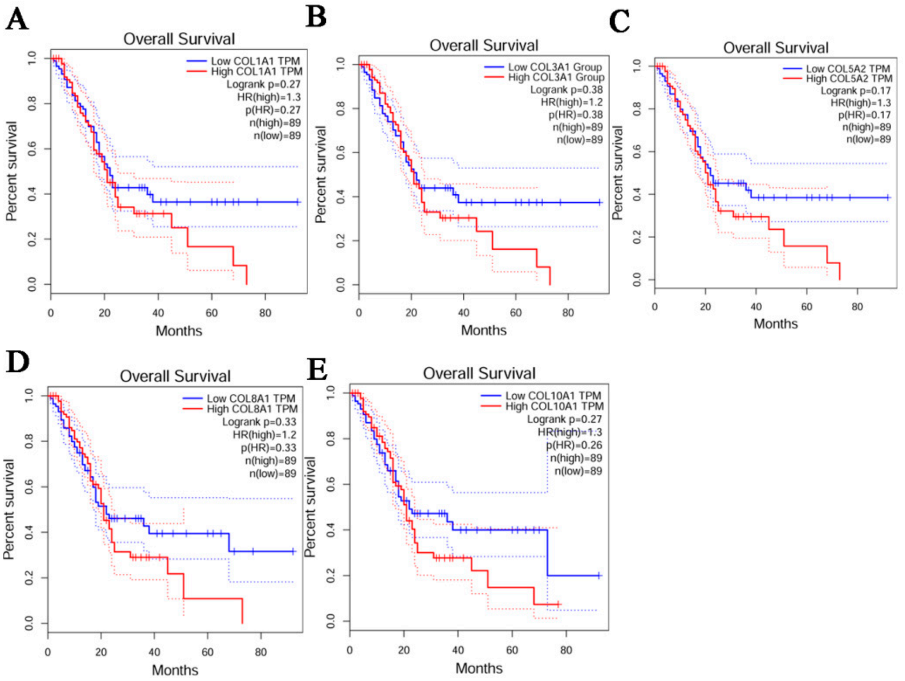

Supplement Figure S1. Survival rates for COL1A1, COL3A1, COL5A2, COL8A1, and COL10A1 high and low expressions in pancreatic cancer.

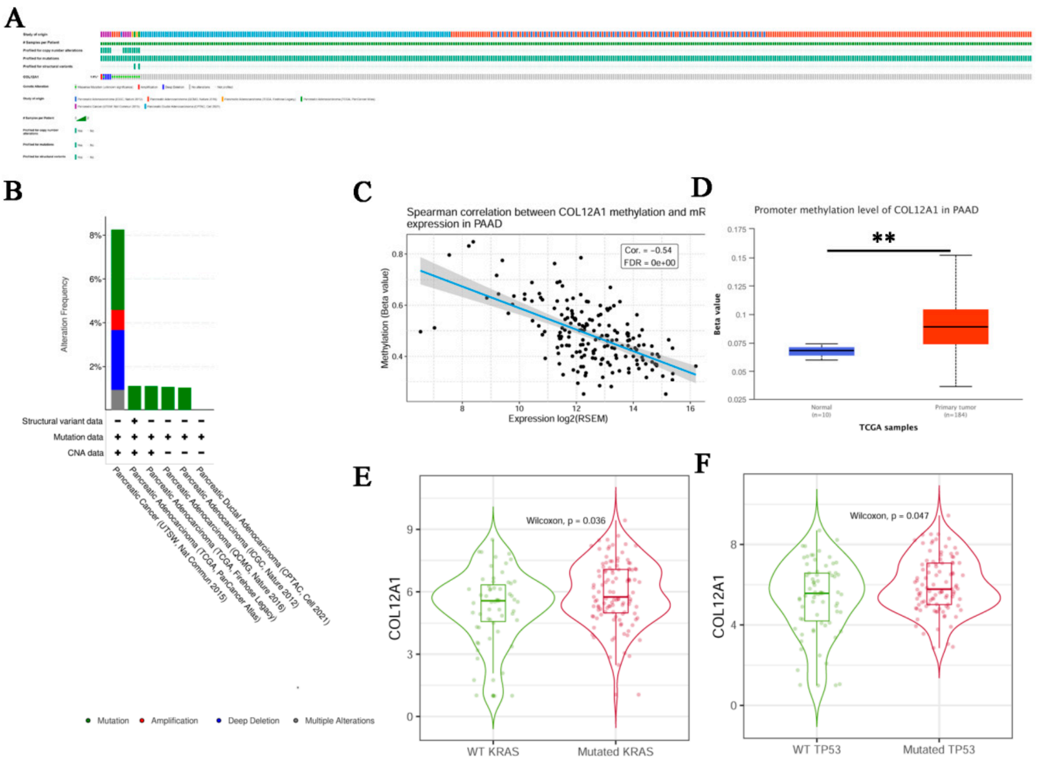

Supplement Figure S2. Genetic Alterations and Mechanism of COL12A1 Regulation. A.

COL12A1 gene alteration in pancreatic cancer. B. Details of COl12A1 gene alteration types in pancreatic cancer; C. Correlation of COL12A1 methylation and mRNA expression; D. Promoter methylation levels of COL12A1 in pancreatic cancer tissue and normal tissue; E. COL12A1 expression in KRAS mutation and wide type pancreatic cancer tissue; F. COL12A1 expression in P53 mutation and wide type pancreatic cancer tissue.

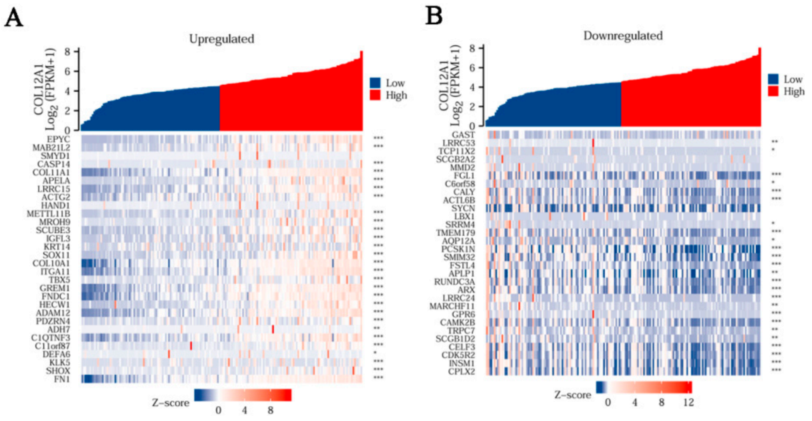

Supplement Figure S3. Represented genes correlated with COL12A1 expression A. Represented genes correlated with COL12A1 expression in downregulated genes. B. Represented genes correlated with COL12A1 expression in upregulated genes.
